# Tests and Procedures for Measuring Endurance, Strength, and Power in Climbing—A Mini-Review

**DOI:** 10.3389/fspor.2022.847447

**Published:** 2022-03-04

**Authors:** Nicolay Stien, Atle Hole Saeterbakken, Vidar Andersen

**Affiliations:** Department of Sport, Food, and Natural Sciences, Faculty of Education, Arts, and Sports, Western Norway University of Applied Sciences, Sogndal, Norway

**Keywords:** reliability, testing, performance, validity, fitness

## Abstract

The interest in climbing is rapidly growing among professional and recreational athletes and will for the first time be included in the 2021 Tokyo Olympics. The sport has also gained increased scientific attention in the past decades. Still, recommendations for testing procedures to predict climbing performance and measure training effects are limited. Therefore, the aim of this mini-review is to provide an overview of the climbing-specific tests, procedures and outcomes used to examine climbing performance. The available literature presents a variety of tests and procedures. While the reliability of some tests has been examined, measures of validity are scarce, especially for climbing-specific endurance tests. Moreover, considering the possible combinations of climbing performance levels, disciplines, and tests, substantial gaps in the literature exist. Vague descriptions of the participants in many studies (e.g., not specifying preferred discipline, performance level, experience, and regular climbing and training volume) further limit the current knowledge and challenge comparisons across studies. Regarding contraction types, dynamic strength- and power-tests are underrepresented in the literature compared to isometric tests. Studies exploring and reporting the validity and reliability of climbing-specific tests are warranted, and researchers should strive to provide a detailed description of the study populations in future research.

## Introduction

In the 2021 Tokyo Olympics, climbing included three disciplines (speed-, lead-, and boulder climbing). Bouldering is performed on low walls (<6 m) with few, difficult, and often highly explosive moves (White and Olsen, 2010), whereas lead climbing is performed on higher walls (10–30 m) and consists of 20 to 50 moves with repeated sub-maximal force generation (Stien et al., 2021a). Speed climbing is performed on a slightly overhanging 15 m wall with a standardized route (Levernier et al., 2020). Success in climbing requires psychological, technical, and physical components (Vigouroux and Quaine, 2006; Baláš et al., 2012; Philippe et al., 2012). Among the physiological requirements, coaches and researchers highlight upper-body strength, power, and endurance as primary factors underpinning performance (MacLeod et al., 2007; Draper et al., 2011; Baláš et al., 2012). Despite noteworthy differences (Fanchini et al., 2013; Ozimek et al., 2017; Stien et al., 2019; Levernier et al., 2020), the three disciplines likely require partly overlapping requirements (Medernach et al., 2016). However, the tests and procedures used to measure these skills vary. Therefore, this mini-review aims to provide an overview of the climbing-specific tests, procedures and outcomes used to assess climbing performance and training effects.

## Methods

A literature search was conducted, including the search terms “Climbing,” “Test,” “Assessment,” “Endurance,” “Strength,” “Force,” “Intermittent,” “Forearm” and “Finger.” Twenty-five relevant studies including climbers and describing at least one experimental testing procedure used to assess the physical characteristics of the study population were included in this mini-review. Please see Supplementary Materials 1, 2 for a more detailed description of the search strategy and screening process.

## Climbing-Specific Endurance Tests

Climbing is characterized by intermittent contractions of the finger flexors. This leads to oxidative and non-oxidative metabolic demands which have been associated with climbing performance (Fryer et al., 2018; Michailov et al., 2018; Giles et al., 2021). The most common endurance tests used to examine these capacities include intermittent or sustained contractions of the finger flexors using climbing-holds (Philippe et al., 2012; Fryer et al., 2015; Michailov et al., 2018; Stien et al., 2019) or handheld dynamometers (Mermier, 2000; Limonta et al., 2015). In addition to finger-specific tasks, number of pull-ups using different holds (Vigouroux et al., 2018), and trunk muscle tests (Saeterbakken et al., 2018; Draper et al., 2021) have been used to examine climbing-related endurance. Recently, testing procedures mimicking climbing have been examined, including motorized climbing ergometers (treadwalls), bouldering, campus board, and lead climbing (Medernach et al., 2015b; Hermans et al., 2017; Baláš et al., 2021; Stien et al., 2021b). Additionally, oxygen-uptake and -saturation have recently been measured as local aerobic capacity of the finger flexors (Baláš et al., 2021). Finally, the critical-force model has recently been introduced to assess the break point of isometric finger flexor work and time to exhaustion (Giles et al., 2021).

### Isometric Sustained Tests

The most frequently applied sustained endurance tests include the bent-arm hang test, finger hang (or dead-hang), and handgrip dynamometers using 40–80% of maximal voluntary contraction (MVC). Of note, only three studies have reported the intraclass correlations (ICC) and coefficients of variation (CV) of endurance tests (Bergua et al., 2018; Fryer et al., 2018; Draper et al., 2021). The reported ICCs and CVs have ranged from 0.881–1.0 and 0.5–18%, likely depending on the climbers' performance level. Bent-arm hang measures time to fatigue hanging from a gym bar with a 90° elbow flexion while keeping the chin above the bar for as long as possible. During the finger hang test, however, the elbows are fully extended, and hold depth varies. Typically, elite climbers have used 10 mm deep rungs, whereas 14–30 mm rungs have been used for intermediate and advanced climbers. Finally, different grip positions used in climbing (slope, pinch, half- and full-crimp) have been examined in the finger hang test with the half crimp being the most frequently used grip (Baláš et al., 2012; Medernach et al., 2015b).

### Intermittent Tests

In the intermittent endurance tests, the arms and/or finger flexors have been examined using handheld or custom-built dynamometers with integrated or connected force cells. The work time has varied from 5 to 10 s with relaxation times between 2 and 5 s, whereas the force threshold has ranged between 40 and 80% of MVC (Vigouroux and Quaine, 2006; MacLeod et al., 2007; Philippe et al., 2012; Michailov et al., 2018; Giles et al., 2021; Rokowski et al., 2021). Furthermore, the testing procedures include uni- and bilateral contraction in addition to extended (180°) (Medernach et al., 2015b) and flexed (90°) elbows (Vigouroux and Quaine, 2006). Of note, both climbing-specific holds with different depths (20–30 mm) and less climbing-specific handheld dynamometers have been used. The only ICC reported was 0.887 using a 23 mm-deep hold with an 8:2 work relaxation ratio using 60% of MVC among advanced climbers (Michailov et al., 2018).

### Climbing and Other Tests

The most specific endurance tests in climbing are climbing to failure tests. Since the route is difficult to standardize (hold size, steepness, distance between holds), re-producible settings have been used. For example, Medernach et al. (2015b) used a 4.1 m high wall with different sized rungs (20–45mm) where the climbers had to maintain a position (4–10 s) before progressing to the next hold. More recently, climbing to failure using a treadwall was introduced and proved suitable for assessing climbing-specific endurance (Baláš et al., 2021). In addition, Stien et al. (2021b) used moves to failure on an overhanging campus board (13 cm separating the 20 mm deep rungs). Importantly, the campus board test only targets the fingers and pulling apparatus, and not the whole body (Stien et al., 2021b). Finally, number of pull-ups has been used as a measure of upper-body endurance and/or strength capacity using 10–80 mm deep holds. Depending on the performance level, decreasing hold depths may target the strength capacity more than deeper holds (Vigouroux et al., 2018).

## Climbing-Specific Strength and Power Tests

The current consensus states that maximal and explosive strength in the fingers and upper-body are crucial factors for climbing performance (Horst, 2016; Sanchez et al., 2019; Saul et al., 2019). However, there is no agreement on how strength in the fingers and upper-body should be assessed. The applied methods vary in hold types, contraction form, body positioning, measuring techniques [e.g., time periods for calculating rate of force development (RFD)], execution (e.g., verbal instructions and duration), and joint angle and number of included joints.

### Dynamometer Tests

Finger strength has been assessed using handheld dynamometers (Baláš et al., 2012; Ozimek et al., 2016). Despite providing a simple and accessible testing method, handheld dynamometer measurements may not reflect climbing performance (Ozimek et al., 2016; Marcolin et al., 2020). Still, handheld dynamometers have been reliable (Baláš et al., 2012; Medernach et al., 2015a), and able to discriminate between climbers and non-climbers (Quaine et al., 2003; Macdonald and Callender, 2011; Limonta et al., 2015; Assmann et al., 2020). Recently, tests that closely mimic the hold types and arm positions in climbing have been implemented (Levernier and Laffaye, 2019; Baláš et al., 2021; Rokowski et al., 2021; Stien et al., 2021a). Using climbing-specific test set-ups rather than handheld dynamometers could be especially important when assessing training effects and comparing different performance levels.

### Isolated Forearm Tests

Typically, finger strength tests include fixating the elbow to potentially exclude force production from the arm- and back muscles (Grant et al., 1996; MacLeod et al., 2007; Marcolin et al., 2020; Stien et al., 2021a). This is usually achieved by positioning the elbow against a surface to restrict any movement, whereas the distance from the surface to the hold is adjusted to allow the finger flexors to exert force in the desired position. The fingers are typically positioned in a half-crimp grip on a climbing hold, likely providing a more sport-specific condition compared to handheld dynamometers (Ozimek et al., 2016; Marcolin et al., 2020). This and similar set-ups have displayed (1) ability to discriminate between performance levels (Grant et al., 1996; MacLeod et al., 2007), and (2) changes in finger strength following a training period (Stien et al., 2021a). Researchers have suggested that climbing-specific maximal strength and RFD tests performed standing on the ground with fixed elbows produced more reliable results (ICC = 0.94) compared to performing the tests with fully extended elbows (ICC = 0.88) (Michailov et al., 2018). However, the results following the extended elbow tests were more strongly associated with climbing performance.

### Isometric Pulling Tests

Recently, researchers have explored tests measuring the force generated by the upper-body pulling apparatus (arms- and back-muscles) (Levernier and Laffaye, 2019; Stien et al., 2021b,c). Such test set-ups might provide a higher climbing-specificity, but at the expense of reliability as the inclusion of more joints could entail a larger variation in results (Stien et al., 2021c). Using an unconstrained, 90° elbow angle, Levernier and Laffaye (2019) demonstrated that maximal strength (CV = 2.9–10.0%) and RFD (CV = 7.8–28.3%) assessed standing and with an open-hand grip were reliable and able to discriminate between novice, skilled, and international climbers. Moreover, the authors assessed different absolute [milliseconds (ms) from the onset of force] and relative calculations of RFD [percentage from the onset (0%) to the peak force output (100%)]. The study concluded that RFD calculated using the first 200 ms (CV = 7.8–16.1%) and 95% of the force curve (CV = 12.6–28.4%) were the most reliable and discriminatory calculations of RFD. In contrast to Levernier and Laffaye (2019) and Stien et al. (2021c) included a bilateral hanging test with a half-crimp grip on a 23 mm rung with a 90° elbow angle. In agreement with Levernier and Laffaye (2019), RFD calculated using longer time scales (≥75% from the onset) were the most reliable and discriminatory measurements and the authors demonstrated CV-values between 10.0 and 31.3% for RFD among advanced-to-elite climbers. Importantly, due to a lack of differences between intermediate and advanced climbers and the high CV values observed for these groups (CV = 20.0–31.3%), Stien et al. (2021c) speculated that the possible difference in RFD was diminished by the very demanding nature of the test. Finally, the findings by Levernier and Laffaye (2019) and Stien et al. (2021c) agree, suggesting that maximal strength could be a more reliable measure than RFD.

#### Isometric Dead-Hang Strength Tests

In two studies, López-Rivera and González-Badillo (2012, 2019) measured maximal finger-strength as the highest extra-weight the participants could maintain for five seconds on a 15 mm hold with extended elbows. López-Rivera and González-Badillo (2012) reported that the test was sufficiently reliable, but they were unable to detect intra- or inter-group differences following eight weeks of fingerboard training. Later, the authors demonstrated significant pre-to-post changes in maximal strength, but no between-groups differences (López-Rivera and González-Badillo, 2019). A high reliability was also reported by Torr et al. (2020) who examined unilateral maximal hangs from a 20 mm rung while using an external unloading of the body mass. The total load (body mass – unloading) that participants could maintain for five seconds displayed excellent reliability between laboratory visits (ICC = 0.91–0.98) and a moderate correlation to climbing performance level (*r* = 0.42–0.50). Albeit unable to provide additional data (e.g., RFD), the test proposed by Torr et al. (2020) presents a sensitive and low-cost method that can be used to monitor intervention effects or to prescribe training loads.

### Dynamic Strength and Power Tests

Finally, dynamic tests focusing more on the upper-body strength than the fingers have been applied (Draper et al., 2011; Laffaye et al., 2014; Ozimek et al., 2016; Levernier et al., 2020; Stien et al., 2021b). For example, Levernier et al. (2020) measured force and velocity during dynamic pull-ups on a gym bar with external loads (0–70% of body mass) and concluded that the test was reliable (*CV* = 1.0–6.6%) and could differentiate between disciplines in higher-elite athletes. Examining 1-RM pull-up on a gym bar, Ozimek et al. (2016) also demonstrated acceptable reliability (*CV* = 7.7%), but noted that the test may lack specificity to climbing. Furthermore, Laffaye et al. (2014) analyzed power output during an arm-jump test from deep jug holds. This test displayed high reliability (*CV* = 4.89%) and could differentiate between intermediate-to-elite climbers. Furthermore, Stien et al. (2021b) measured maximal campus board reach. Albeit able to detect within- and between-groups differences, the authors did not report the reliability of the test. In comparison, Draper et al. (2011) used a power-slap test from large jug holds and measured the maximal reach. This test was reliable (ICC = 0.95–0.98) and related to climbing ability (*r* = 0.69–0.73).

## Discussion

Climbing performance is measured using graded boulders or routes which categorize the performance levels (Draper et al., 2016). However, concurrent improvements in climbing-tests and -performance are poorly described in the literature (Hermans et al., 2017), whereas the association between climbing-specific tests and climbing performance has been examined (Baláš et al., 2012; Fryer et al., 2015; Laffaye et al., 2016). Several climbing-specific tests and procedures have not been validated and reliability measurements of the tests are rarely reported. Furthermore, the current findings indicate that reliability data are more frequently reported than validity data. This presents a gap in the knowledge which should be addressed in future research. In addition, and despite the differences in climbing-style and physiological requirements (Fanchini et al., 2013; Fryer et al., 2017; Stien et al., 2019), specific tests for individual disciplines do not exist.

The available literature is challenged by the vast variety of applied endurance-, strength-, and power tests (Ozimek et al., 2016; Michailov et al., 2018; Levernier and Laffaye, 2019; Torr et al., 2020; Stien et al., 2021c). For example, this review revealed 13 trials that had implemented the intermittent forearm endurance test, and these provided nine different combinations of work-to-rest ratios and force thresholds (Table 1). Moreover, the study populations in various investigations range from non-climbers to higher-elite athletes. Hence, a very small portion of the possible climbers-and-tests combinations have been thoroughly examined. It is paramount that researchers strive to provide detailed descriptions of the included population and validity, reliability, and sensitivity measures of the tests applied in future research.

**Table 1:** Climbing-specific endurance applied in the available literature.

**References**	**Subjects**	**Performance level**	**Test procedures**	**Outcomes**	**Reliability**	**Correlation with performance**
**Isometric tests with sustained/continuous force generation**						
Mermier (2000)	44 •	Lower-grade to elite	Bent-arm hang: The subjects hang with a 90° elbow angle using the biggest holds on a climbing fingerboard. Grip endurance: dominant hand was used to measure the time maintaining 50% of MVC using a handheld dynamometer.	Time to fatigue	•	*r* = 0.798
Baláš et al. (2012)	205 •	Lower-grade to higher-elite	Bent-arm hang: The subjects hang with overhand grip (shoulder width) in a bar (2.5 cm wide) in a pull-up position with chin above the bar. Bilateral finger-hang with fully extended elbows and with four fingers open or crimp grip on a 2.5 cm ledge.	Time to fatigue	•	Bent-arm hang: r^2^ = 0.49–0.64 Finger-hang: r^2^ = 0.66–0.76
Limonta et al. (2015)	11 •	Elite and higher-elite	A handgrip ergometer was used to measure time to fatigue using 80% of MVC (± 5%).	Time to fatigue	•	•
Medernach et al. (2015a)	23 BC	Advanced	Bi-lateral finger-hangs using: (1) half crimp grip on a 19 mm deep edge, (2) pinch grip, (3) slope grip and (4) 30 mm-deep ledge crimp grip (Alien Fingerboard).	Time to fatigue	•	•
Ozimek et al. (2016)	14 •	Advanced and elite	Finger hang were the subjects hang from a 4 cm ledge with a half-crimp grip.	Time to fatigue	•	•
Bergua et al. (2018)	40 LC	Advanced and elite	Finger hang tests using open- and half crimp were conducted on 1) a 14 mm ledge and 2) the minimum ledge depth the subjects could hang for 40 s.	1) Time to fatigue 2) Ledge dept.	1) ICC = 0.91-0.99 2) ICC = 0.89-1.00	•
López-Rivera and González-Badillo (2019)	26 LC	Elite	Finger hang from an 11 mm deep ledge using a half crimp grip.	Time to fatigue	•	*r* = 0.62
Fryer et al. (2018)	29 LC 9 NC	Intermediate to elite	Duration of sustained arm flexors contraction at 40% of MVC using a fingerboard with an open crimp grip.	Duration and force time integral [0.4 MVC x contraction (s) x force (N)]	MVC: CV = 0.5%	•
Draper et al. (2021)	132 •	Lower-grade to elite	Bent-arm hang: The subjects hang with overhand grip (shoulder width) on a bar (2.5 cm wide) in a pull-up position with chin above the bar. Finger-hang; the subjects hang with an open crimp hold using a 30 mm deep rung	Time to fatigue	Bent-arm hang: ICC = 0.894, CV: 18% (12-32) Finger-hang: ICC = 0.881, CV: 15% (11-24)	•
Philippe et al. (2012)	12 • 12 NC	Elite and higher elite	Unilateral sustained finger flexors test to failure using 40% of MVC on a 22 mm deep wooden hold.	Time to fatigue, force integral	•	•
Baláš et al. (2021)	22 LC	Intermediate and advanced	Unilateral sustained finger flexors test using 60% of MVC on a 23 mm deep wooden hold.	Time to fatigue	•	*r* = 0.560
Rokowski et al. (2021)	14 LC	Advanced to higher elite	Unilateral sustained force production (60% of MVC) to failure on a 23 mm deep wooden hold. Performed standing with a near full elbow extension.	Time to fatigue and force-time integral relative to BM	•	Time to fatigue: *r* = −0.261 Integral: *r* = 0.54
**Isometric, intermittent force generation to failure**						
Michailov et al. (2018)	22 •	Intermediate and advanced	Unilateral intermittent finger flexor endurance using a 23 mm deep climbing hold with an open-finger grip position (thumb as disengaged). The work relaxation ratio was 8:2 using 60% of MVC	Time to fatigue	ICC = 0.887 *n* = 9 included in reliability test	•
MacLeod et al. (2007)	11 LC	Intermediate and advanced	Unilateral intermittent finger flexor test using an open crimp grip with a 90° angle of the elbow and shoulder using 40% of MVC with an 8:2 work relaxation ratio	Time to fatigue	•	•
Medernach et al. (2015a)	24 BC	Advanced	Bi-lateral intermittent isometric test with a 30- mm deep crimp grip (Alien Fingerboard) fixed at 120° beyond vertical. The work relaxation ratio was 8:4 hanging (i.e., body-mass).	Time to fatigue	•	•
Giles et al. (2021)	11 LC	Advanced to higher-elite	Bi-lateral intermittent finger hang test on a 20 mm-deep edge (Lattice training rung) using half-crimp hold. The work relaxation ratio was 7:3 using 80%, 60%, and 45% of MVC.	Time to fatigue and time to critical force	•	•
Stien et al. (2019)	16 BC 15 LC	Advanced	Bi-lateral intermittent finger flexor test in a seated position with shoulder fully adducted and with a 90° elbow flexion. A 23 mm-deep edge was used with an open crimp grip and 70% of MVC in a 7:3 work relaxation ratio.	Time to fatigue	•	•
Vigouroux and Quaine (2006)	9 LC	Elite and higher-elite	Unilateral intermittent finger flexor test in a seated position with 45° shoulder abduction and 90° elbow flexion. The work relaxation ratio was 5:5 using 80% of MVC.	Time to fatigue	•	•
Baláš et al. (2021)	22•	Intermediate to advanced	Unilateral intermittent finger flexors test using 60% of MVC with fully extended elbow on a wooden hold with 23 mm dept. The work relaxation ratio was 8:2.	Time to fatigue, oxygen saturation.	•	•
Philippe et al. (2012)	12 • 12 NC	Elite and higher elite	Unilateral intermittent finger flexors test using 40% of MVC on a 22 mm deep wooden hold. The work relaxation ratio was 10:3.	Time to fatigue, force integral	•	•
Quaine et al. (2003)	20 LC	Novice and elite	Unilateral intermittent finger flexor test on a 20 mm deep hold performed in a seated position with 45° shoulder abduction and 90° elbow flexion. Tested at 80% of MVC with a work relaxation ratio of 5:5.	Time to fatigue	•	•
Baláš et al. (2021)	22 LC	Intermediate and advanced	Unilateral intermittent finger flexors test using 60% of MVC on a 23 mm deep wooden hold. The work relaxation ratio was 8:2.	Time to fatigue	•	*r* = 0.486
Rokowski et al. (2021)	14 LC	Advanced to higher elite	Unilateral intermittent force production (60% of MVC) to failure on a 23 mm deep wooden hold. Performed standing with a near full elbow extension. The work relaxation ratio was 8:2.	Time to fatigue Force-time integral relative to BM	•	Time to fatigue: *r* = −0.268 Integral: *r* = 0.191
**Climbing tests**						
Medernach et al. (2015b)	24 BC	Advanced	Climbing to failure on a 4.1 m high wall (120° overhang) with four grips (20, 30, 45, and 45 mm-deep ledges. Climbers had to maintain an isometric position for 4, 6, 8, and 10 sec) before moving to the next ledge.	Inability to continue climbing	•	•
Hermans et al. (2017)	30 •	Lower-grade and intermediate	An 18 m route with progressively increasing difficulty was used. The route included 43 holds and points were given for each handhold passed. Top rope was used during testing	Numbers of handholds passed	•	•
Baláš et al. (2021)	22 •	Intermediate and advanced	Climbing to failure on 3.8 m treadwall with 14 hand moves graded 8 on the IRCRA scale with a speed of 9 m/min with increasing steepness (-5°) every minute. A sustained test to fatigue	Time to fatigue, heart rate, VO_2_peak, ventilation x min^−1^	•	•
Stien et al. (2021b)	16 •	Advanced and elite	Numbers of moves on a campus board with single arm moves up- and downwards. The board was overhanging (15°) and 13 cm separated the 20 mm-deep ledges.	Numbers of moves to fatigue	•	•
Schöffl et al. (2006)	28 LC	Elite	Climbing to failure on a treadwall.	Climbing time to failure	Between-sessions correlation: *r* = 0.99	•
Limonta et al. (2018)	13 LV	Advanced and elite	Climbing to failure on a treadwall.	Oxygen uptake and workload	•	•
**Other tests**						
Vigouroux et al. (2018)	10 •	Advanced to higher-elite	Numbers of pull-ups using 10, 14, 18, 22, 80 mm deep holds and a 2.5cm gym bar. The climbers were instructed to conduct the repetitions with maximal effort.	Number of pull-ups	•	•
Saeterbakken et al. (2018)	19 •	Advanced	Hanging vertically from a 6 cm beam and placed one foot on a chip 185 cm above the ground and the participant‘s body length in the horizontal direction. Maintained position for one second before lowering the body.	Numbers of completed repetitions	•	•
Draper et al. (2021)	132 •	Lower-grade to elite	Prone plank with the elbows bent at 90° and placed directly beneath the shoulders. The body had to form a straight line from head to feet.	Time to fatigue	•	•

*IRCRA, International Rock Climbing Research Association; BC, Boulder climbers; LC, lead climbers; MVC, maximal voluntary contraction; CV, coefficient of variation; ICC, intraclass correlation; r, correlation coefficient; •, not reported*.

*Performance level calculated using the grouping proposed by Draper et al. (2016)*.

Although researchers may argue that some test set-ups are superior to others regarding reliability or specificity to climbing, the current available evidence could be too fragmented to support either position. Moreover, it is possible that choosing to optimize conditions for either specificity or validity will come at the cost of the other. For example, complex tests may provide conditions that mimic climbing more closely but could also increase the difficulty of reproducing similar results. Importantly, the complex nature of climbing renders it challenging to argue which test set-up is more climbing-specific. More descriptive studies such as biomechanical- (Cha et al., 2015), motion- (White and Olsen, 2010), and workload-analyses (Michailov, 2014) in climbing are needed to provide a basis for test recommendations.

Currently, reliability data has only been reported for a handful of protocols. Isometric endurance tests with sustained force generation (e.g., finger-hang or bent-arm hang) may be easy to conduct, but do not mimic the locomotion in climbing. Isometric intermittent tests to failure have a greater ecological validity, but there is no consensus in work-relaxation ratio, force threshold, hold size, or grip position. In addition to the varying work-to-rest ratios and force thresholds, different hold depths (10–30 mm), hold types (jug, gym bar), and grip positions (half-crimp or open-hand) have been used. The more promising tests are climbing to fatigue tests using reproducible routes or standardized walls (Medernach et al., 2015b; Baláš et al., 2021; Stien et al., 2021b). However, these tests suffer from limited research and the findings may not be generalizable to other disciplines or performance levels.

Based on the previously reported reliability data, one could speculate that hold size greatly influences the reliability of a test, regardless of task complexity and contraction form. For example, some of the smallest CV-values reported for power and isometric strength (1.0–6.6%) have been collected from tests that used either jug holds (Laffaye et al., 2014; Stien et al., 2021b) or a gym bar (Levernier et al., 2020). For maximal strength, Stien et al. (2021b) reported a 1.1% CV using jug holds, compared to 4.7% using a 23 mm rung. Shallower holds (~10–20 mm) have displayed CV-values between 7.8 and 31.3% (López-Rivera and González-Badillo, 2012; Ozimek et al., 2016; Stien et al., 2021c). Indeed, the fingers are likely the weakest link in the pulling apparatus and hold depth influences the biomechanical arm action during pulling movements (Vigouroux et al., 2018). Future studies should identify whether the climbing-specificity of a test is compromised by using large holds, or if large holds can maintain validity while increasing reliability.

Finally, dynamic tests are underrepresented in the literature (Table 2). Although climbing is characterized by isometric contractions of the finger flexors, the movements in the elbows and shoulders are often dynamic to produce vertical propulsion. Hence, one could argue that future research should focus more on dynamic strength in the upper-limbs of climbers, in addition to isometric strength in the finger flexors. Indeed, investigations using dynamic tests have demonstrated that such test set-ups are (1) reliable, (2) able to differentiate between performance levels and disciplines, and (3) sensitive enough to detect within- and between-groups differences following a training intervention (Laffaye et al., 2014; Ozimek et al., 2016; Levernier et al., 2020; Stien et al., 2021b).

**Table 2:** Strength and power tests applied in the available literature.

**References**	**Subjects**	**Performance level**	**Test procedures**	**Outcomes**	**Reliability**	**Correlation with performance**
**Isometric dynamometer tests**						
Baláš et al. (2012)	205 LC	Advanced and elite	Handheld dynamometer with 180° elbow angle. At least 2 s hold	MVC	•	r^2^ = 0.10–0.11
Ozimek et al. (2016)	14 •	Advanced to higher-elite	Handheld dynamometer with a 180° elbow angle.	MVC	CV = 9.7–10.0	•
Grant et al. (1996)	10 NC 20 LC	Recreational and elite	Table-mounted dynamometer with 90° elbow angle and a half-crimp grip. Force measured during 2 s maximal effort.	MVC	•	•
Marcolin et al. (2020)	34 LC 15 NC	Intermediate to higher-elite	Table-mounted dynamometer with 90° elbow angle and a half-crimp grip on a 22 mm ledge. Force measured during 2 s maximal effort.	MVC	•	*r* = 0.60
MacLeod et al. (2007)	11 LC 9 NC	Intermediate and advanced	Table-mounted dynamometer with 90° elbow angle and a half-crimp grip. Force measured during 2 s maximal effort.	MVC	•	*r* = 0.706
Fanchini et al. (2013)	10 LC 10 BC 10 NC	Advanced and elite	Seated, using a custom-built dynamometer during 3 s hold with a 180° elbow angle.	MVC RFD_peak_	ICC > 0.90	•
Michailov et al. (2018)	22 •	Intermediate and advanced	Standing, using a wall-mounted dynamometer. Force measured during with 90° and 180° elbow angles.	MVC	90° elbow: ICC = 0.941 180° elbow: ICC = 0.878	90° elbow: *r* = 0.45–0.46 180° elbow: *r* = 0.61–0.74
Stien et al. (2021a)	14 •	Intermediate and advanced	Table-mounted dynamometer using half-crimp on a 23 mm rung. Elbow constrained in 90°.	MVC	•	•
Philippe et al. (2012)	12 • 12 NC	Elite and higher elite	Table-mounted dynamometer with 90° elbow angle and a half-crimp grip on a 22 mm ledge. Maximal force reached in five seconds.	MVC	•	*r* = 0.839
Baláš et al. (2021)	22 LC	Intermediate and advanced	Unilateral hangs on 23 mm ledge with built-in force sensor. Had to hold for 5 s.	MVC	•	*r* = 0.552
Levernier and Laffaye (2019)	22 BC 9 NC	Advanced to higher elite	Wall-mounted dynamometer with unconstrained 90° elbow angle using open hand and half crimp grips on a 10 mm hold. RFD collected at 50, 100, and 200 ms from onset of force, as well as at 95% of peak force.	RFD MVC	RFD: ICC = 0.58–0.98 CV = 7.8–28.4% MVC: ICC = 0.94–0.99 CV = 2.6–5.9%	•
Rokowski et al. (2021)	14 LC	Advanced to higher elite	Unilateral maximal force production on a 23 mm deep wooden hold. Performed standing with a near full elbow extension. Five seconds time window available for force production.	Peak force	•	*r* = 0.241
**Isometric fingerboard tests**						
López-Rivera and González-Badillo (2012)	9 LC	Elite and higher-elite	Dead-hang using 15 mm ledge with straight arms and half-crimp grip. Had to hold for 5 s.	Maximal extra-load (kg)	CV = 7.8% ICC = 0.96	•
Torr et al. (2020)	229 •	Intermediate-to-higher elite	Unilateral hangs on 20mm ledge with de-load. Had to hold for 5s.	Maximal total load	ICC = 0.91–0.98	*r* = 0.42–0.50
Ozimek et al. (2016)	14 •	Advanced to higher-elite	Dead-hang using 25 mm ledge and a half-crimp grip. Had to hold for 3 s.	Maximal total-load	CV = 22.9%	•
Stien et al. (2021c)	57 LC	Intermediate to elite	Isometric pull-up on 23 mm ledge using a half-crimp and 90° elbow angle	MVC RFD	CV = 9–20% ICC = 0.88–0.99	•
**Dynamic tests**						
Levernier et al. (2020)	11 BC 8 LC 5 SC	Higher-elite	Two pull-ups with 0, 30, 45, 60, and 70% BM extra-load in random order. Vertical velocity measured with accelerometer attached to the waist belt.	Force Velocity	CV = 1.0–6.6%, ICC = 0.84–0.99	•
Laffaye et al. (2014)	34 •	Intermediate to elite	Arm-jump board test from jug hold. Power output measured with accelerometer.	Power	CV = 4.89%, ICC = 0.976	•
Ozimek et al. (2016)	14 •	Advanced to higher-elite	1RM pull-up with extra-load performed on a gym bar.	Maximal total load	CV = 7.7%	•
Stien et al. (2021b)	17 LC	Advanced and elite	Maximal reach with one hand performed on a 15° overhanging campus board using 20 mm rungs. 13 cm between ledges.	Number of rungs reached	•	•
Draper et al. (2011)	38 LC	Novice to elite	Maximal reach (powerslap) with one hand performed on a custom board using jug holds.	Reach (cm)	ICC = 0.95–0.98	*r* = 0.69–0.73

*IRCRA, International Rock Climbing Research Association; BC, Boulder climbers; LC, Lead climbers; SC, speed climbers; NC, non-climbers; RFD, rate of force development; RFD_peak_, RFD calculated using the steepest portion of the force curve; MVC, maximal voluntary contraction; s, seconds; BM, body mass; CV, coefficient of variation; ICC, intraclass correlation; r, correlation coefficient; •, not reported*.

*Performance level calculated using the grouping proposed by Draper et al. (2016)*.

Some recommendations can be made based on the finding of this mini-review. Importantly, the scarcity of relevant studies should be considered when interpreting the results, as well as the conflicting findings between studies. For isolated endurance tests, the force-time integral might be more useful compared to simply reporting the total work time (Rokowski et al., 2021). Moreover, the time to fatigue during sustained endurance tests has displayed moderate-to-strong correlations with red-point climbing performance, whereas the few studies that examined intermittent tests reported weak correlations with climbing performance (Baláš et al., 2021; Rokowski et al., 2021). For strength, more reliable results might be achieved by using isometric dynamometer tests (CV ≤ 10%) compared to fingerboard tests (CV ≤ 22.9%). The validity will likely differ depending on the test set-up (e.g., elbow angle, body positioning, and grip type) and study population (e.g., performance level or preferred discipline), but in general the seated, 90° constrained elbow set-up displayed the highest correlations with climbing performance (*r* = 0.60–0.84) (Philippe et al., 2012; Marcolin et al., 2020). Dynamic upper-body strength tests (e.g., pull-up) also revealed a high reliability (CV ≤ 7.7%), but such tests could potentially lack specificity to climbing compared to tests focusing on the finger flexors (Ozimek et al., 2016). Finally, high intensity, upper-body tests (i.e., powerslap and campus board reach) were investigated in two studies (Draper et al., 2011; Stien et al., 2021b) and displayed a strong relationship with climbing performance (*r* = 0.69–0.73).

Establishing reliable and valid testing procedures is essential for the field of climbing research. Today, no consensus exists regarding preferred sport-specific performance assessments. This study provides a brief overview of the applied endurance-, strength-, and power-tests, and highlights gaps in the literature. The findings of this mini-review revealed that numerous approaches to measuring climbing-related performance have been applied, but few have reported the reliability and validity of the tests. Hence, the current knowledge is fragmented as very few findings have been re-tested in subsequent studies with similar methodology. Moreover, poor descriptions of populations challenge performance level- and discipline-specific testing recommendations. Importantly, the pioneer work by Draper et al. (2016) needs to be acknowledged for first providing a numerical scale, making it possible to compare climbing performance across continents, and more recently attempting to establish a test battery (Draper et al., 2021). Hopefully, this mini-review will provide a useful overview of the scientific literature and inspire researchers to work toward agreeing upon common tests and procedures.

## Author Contributions

NS and AS wrote the first draft of the article and extracted the relevant data from the included studies. VA reviewed the data extraction and settled any disagreements between NS and AS. All authors contributed to the conceptualization and methodology. All authors provided critical reviews of the paper.

## Conflict of Interest

The authors declare that the research was conducted in the absence of any commercial or financial relationships that could be construed as a potential conflict of interest.

## Publisher's Note

All claims expressed in this article are solely those of the authors and do not necessarily represent those of their affiliated organizations, or those of the publisher, the editors and the reviewers. Any product that may be evaluated in this article, or claim that may be made by its manufacturer, is not guaranteed or endorsed by the publisher.
