# Supplementary material for: Tests and Procedures for Measuring Endurance, Strength, and Power in Climbing—A Mini-Review
Source: Front Sports Act Living. 2022 Mar 4;4:847447. doi: 10.3389/fspor.2022.847447 (PMC8931302; doi:10.3389/fspor.2022.847447)
Supplement: Supplementary file 1 [file Data_Sheet_1.docx]

**Search strategy and screening process**

A literature search was performed in PubMed and Web of Science for articles including climbers and describing at least one experimental testing procedure used to assess the physical characteristics of the study population. This review focused on strength and endurance parameters, whereas studies reporting only on flexibility or psychological outcomes were excluded. The following search term was used: (Climbing OR Climbers) AND (Test OR Assessment) AND (Endurance OR Strength OR Force OR Intermittent OR forearm OR Finger) NOT (elderly OR stair OR mice OR ladder OR running OR cycling). This search provided a total of 335 results, of which 308 remained after removing duplicates (Figure 1). NS reviewed the titles and deemed 31 articles relevant to the topic. After reading the full-texts, six were removed due to not including climbers (n=2) or not testing climbing-specific strength, power, or endurance (n=4). Thus, 25 articles were included in this review (Tables 1 and 2). NS and AHS independently extracted the data from the studies before the datasets were merged. Any disagreements between the two reviewers were settled by discussion or by including a third reviewer (VA) for consulting.
