# Supplementary material for: Tests and Procedures for Measuring Endurance, Strength, and Power in Climbing—A Mini-Review
Source: Front Sports Act Living. 2022 Mar 4;4:847447. doi: 10.3389/fspor.2022.847447 (PMC8931302; doi:10.3389/fspor.2022.847447)
Supplement: Supplementary file 2 [file Data_Sheet_2.docx]

Records identified:

(n = 335)

Duplicate records removed

(n = 27)

**Identification**

Records screened:

(n = 308)

Records excluded:

(n = 280)

**Screening**

Reports excluded:

Not including climbers (n = 2)

Not testing performance parameters (n = 4)

Reports assessed for eligibility:

(n = 28)

Included from other sources:

(n = 3)

**Included**

Studies included in review:

(n = 25)

**Figure 1:** Flow chart illustrating the search strategy and screening process.
